# Determinants of uncontrolled hypertension among clients on anti-retroviral therapy in Kadoma City, Zimbabwe, 2016

**DOI:** 10.1186/s40885-017-0070-4

**Published:** 2017-07-04

**Authors:** Pamela Nyaradzai Magande, Daniel Chirundu, Notion Tafara Gombe, More Mungati, Mufuta Tshimanga

**Affiliations:** 1 Department of Community Medicine, University of Zimbabwe, Harare, Zimbabwe; 2 Kadoma City Health Department, Kadoma City, Zimbabwe

**Keywords:** Uncontrolled hypertension, ART, Kadoma

## Abstract

**Background:**

Clients on anti-retroviral therapy (ART) are living longer and have risk of hypertension. Side effects of medicines and aging increase this risk. Hypertension prevalence among clients on ART in Kadoma City was estimated to be 30% in 2015. Of these, 61% had uncontrolled hypertension. This was high compared to 46% of hypertensives in the general population who had uncontrolled hypertension. We determined factors associated with uncontrolled hypertension among clients on ART.

**Methods:**

A 1.1 unmatched case control study was conducted. Interviews, anthropometric measurements and record reviews were to collect data on demography and medical history. Epi Info 7 was used for univariate, bivariate analysis and logistic regression.

**Results:**

One hundred and fifty-two cases and 152 controls were recruited into the study. Adding salt to dishes regularly aOR = 5.69 (3.19–10.16), body mass index (BMI) above 25 kg/m^2^ aOR = 2.81 (1.60–4.91) and history of elevated blood pressure in previous year aOR = 2.34 (1.33–4.13) were independent risk factors. Independent protective factors were duration more than 2 years since HIV diagnosis aOR = 0.58 (0.35–0.95), duration less than 5 years since hypertension diagnosis aOR = 0.50 (0.30–0.83) and walking or cycling as a means of transport aOR = 0.27 (0.16–0.48).

**Conclusion:**

Adding salt to dishes regularly, BMI above 25 kg/m^2^, history of elevated blood pressure in the previous year, duration more than 2 years since HIV diagnosis, duration less than 5 years since hypertension diagnosis and walking or cycling as a means of transport were independently associated with uncontrolled hypertension. Health education on lifestyle changes like walking and cycling as transport and dietary modification such as salt intake reduction were recommended.

## Background

Hypertension and Human Immunodeficiency Virus (HIV) infection are chronic conditions which are asymptomatic and manageable in early stage [1]. The interaction between hypertension and HIV is complex. Renal insufficiency due to HIV infection contributes to secondary hypertension [2–4]. The HIV infected on ART live longer and gain weight. Weight gain is a risk factor of hypertension [1]. The weight gain can be explained in two ways. The HIV infected when on ART are less susceptible to opportunistic infections that cause malabsorption syndromes. Weight gain can also be a side effect of ART medicines.

ART medicines such as ritonavir-boosted lopinavir (protease inhibitors), efavirenz and tenofovir are associated with hypertension. This is mediated through their side effects. Studies show that insulin resistance precedes lipodystrophy. The slowest step in glucose metabolism transport mediated by glucose transporter -4 (GLUT-4) in muscle and fat. This process is worsened by protease inhibitors [5, 6]. This results in metabolic syndrome and eventually hypertension. Tenofovir causes renal tubular toxicity resulting in renal dysfunction and eventually hypertension when there is pre-existing renal disease [7–9].

Globally, hypertension caused approximately 12.8% of all deaths in 2012. This accounted for 3.7% disability adjusted life years (DALYS) in the same year [10]. This is expected to increase due to ageing and population growth [11]. Africa has the highest prevalence of hypertension worldwide. In 2012, the prevalence was reported to be 46% [10]. This is higher than the global average which is currently 40% [11]. According to the Zimbabwe STEPwise survey [12], hypertension prevalence was 25% among adults. No current survey based data exists for Zimbabwe. Hypertension prevalence increased by 40% between 2013 and 2015 in Kadoma City [13].

At the end of 2014, 36.9 million people were living with HIV worldwide. Sub-Saharan Africa accounts for 70% of people living with HIV worldwide [14]. In Zimbabwe, approximately 1.6 million people were living with HIV in 2014. Of these, 52% adults were on ART [15]. In Kadoma City, an estimated 4, 430 people were on ART in 2015 [13]. Although all hypertensives are at risk of uncontrolled hypertension, those who are HIV positive and on ART have higher risk [1–3, 16–18]. Hypertension prevalence of hypertension among clients on ART was estimated to be 30% at the end of 2015. Of these, 61% had uncontrolled hypertension. This was high compared to 46% of hypertensives in the general population with uncontrolled hypertension. We determined factors associated with uncontrolled hypertension among clients on ART in Kadoma City.

## Methods

A 1:1 unmatched case control study was conducted. A case was defined as a client registered and receiving care at Rimuka Integrated HIV and Tuberculosis Centre (IHTC) with uncontrolled hypertension. A control was defined as a client registered and receiving care at Rimuka IHTC with controlled hypertension. Uncontrolled hypertension was defined as systolic blood pressure greater than 140mmHG and or diastolic blood pressure > 90mmHG as defined in the JNC 7 report [19].

Based on a study by Mutede et al. [1], assuming 80% power, 95% confidence interval and a case to control ratio of 1:1, with a prevalence of hypertension among clients on anti-retroviral therapy (ART) of 34.9% and an odds ratio of 2.0 for those who had BMI > 25 kg/m^2^, using Fleiss formula, the calculated sample size was 147 cases and 147 controls.

Hypertensive clients on ART who came to IHTC for reviews during the study period were recruited until the sample size was reached. Structured questionnaires were used to collect data on demographics and factors associated with uncontrolled hypertension. The Zimbabwe STEPwise survey 2005 and the World Health Organization Hypertension questionnaires were adapted to make questionnaires [12, 20]. Anthropometric measurements were conducted as described in the Zimbabwe STEPwise survey [12].

Blood pressure measurements were done using an electronic sphygmomanometer calibrated against a standard mercury sphygmomanometer. At least two readings were made for each client. The time between readings was at least two minutes. The presence of a difference of 10 mmHg or more between the first two readings warrantied a third reading. The final record was the mean of two readings with less than a 10mmHG difference. Those with systolic blood pressure >140 mmHg and or diastolic blood pressure >90 mmHg while on treatment were classified as having uncontrolled hypertension according to the JNC 7 report and the CDC definitions. The ascertainment of whether a client is truly a case was done based on the previous two or three blood pressure readings as recorded in the medical records. Adherence to medicines was measured by way of pill counts and client reports.

Medical records reviewed to collect data on medical history. Epi Info 7™ (CDC, 2012) was used to perform univariate and bivariate analysis. To identify independent factors, backward logistic regression using the same software was performed. Adjusted odds ratios (aOR) were then reported after the logistic regression. All variables with a *p* value <0.05 were considered statistically significant.

## Results

### Demographic characteristics

One hundred and fifty-two cases and 152 controls were recruited. The cases and the controls were comparable with regards to age group, marital status, education and employment (Table 1). The respondents were also comparable with regards to the medicines they were taking (Table 2).

**Table 1:** Demographic characteristics of the hypertensive clients on ART, Kadoma City, 2016

Variable		Uncontrolled hypertension		*p value*
		Yes n(%)	No n(%)	
Age in years				
	≤30	10 (6)	12 (7)	0.29
	31–40	19 (13)	27 (18)	
	41–50	61 (40)	71 (47)	
	51–60	45 (30)	36 (24)	
	61–70	15 (10)	4 (3)	
	71–80	2 (1)	2 (1)	
	Median age (Q_1_=; Q_3_=)	49 (Q_1_ = 43; Q_3_ = 54)	45 (Q_1_ = 43; Q_3_ = 52)	
Sex				
	Female	94 (62)	110 (72)	0.051
	Male	58 (38)	42 (28)	
Marital Status				
	Married	81 (53)	80 (53)	0.98
	Widowed	31 (20)	36 (24)	
	Divorced	28 (18)	22 (14)	
	Separated	9 (6)	8 (5)	
	Single	4 (3)	5 (3)	
	Cohabiting	0	1 (1)	
Education				
	Primary	31 (20)	33 (22)	0.14
	Secondary	106 (70)	115 (76)	
	Tertiary	12 (8)	3 (2)	
	None	3 (2)	1 (1)	
Employment				
	Manual	80 (53)	87 (57)	0.84
	Sedentary	67 (44)	56 (37)	
	None	5 (3)	9 (6)

**Table 2:** Pharmacological management of hypertensive clients on ART, Kadoma City, 2016

Medicine		Uncontrolled hypertension		*p value*
		Yes n(%)	No n(%)	
Anti-hypertensives	Hydrochlorothiazide	100 (66)	114 (75)	0.08
	Atenolol	17 (13)	18 (12)	
	Nifedipine	13 (9)	15 (10)	
	Amlodipine	12 (8)	2 (1)	
	Captopril	5 (3)	2 (1)	
	Other	3 (1)	1 (1)	
Anti-retrovirals	Tenofovir/Lamivudine/Efevirenz	144 (95)	144 (95)	0.99
	Tenofovir/Lamivudine/Atazanavir	5 (3)	6 (4)	
	Zidovudine/Lamivudine/Atazanavir	2 (1)	2 (1)	
	Tenofovir/Lamivudine/Nevirapine	1 (1)	0	

### Physical measurements

The cases and the control were comparable with regards to weight and height but not with regards to blood pressure (Table 3).

**Table 3:** Physical measurements for hypertensive clients on ART, Kadoma City, 2016

Variable	Uncontrolled hypertension		*p value*
	Yes n(%)	No n(%)	
Systolic blood pressure (+/-SD)	151 (+/-15.46)	118 (+/-8.91)	<0.05
Diastolic blood pressure (+/-SD)	92 (+/-9.14)	75 (+/-7.05)	<0.05
Height in cm			
≤ 150	4 (3)	3 (2)	0.68
151–160	47 (31)	62 (41)	
161–170	82 (54)	75 (49)	
170–180	19 (12)	12 (8)	
Median height in cm (Q_1_=; Q_3_=)	164 (Q_1_ = 160; Q_3_ = 169)	162 (Q_1_ = 158; Q_3_ = 168)	
Weight in kg			
≤ 50	8 (5)	27 (17)	0.52
51–60	35 (23)	54 (36)	
61–70	51 (34)	43 (28)	
71–80	37 (24)	16 (11)	
81–90	13 (9)	10 (7)	
91–100	8 (5)	2 (1)	
Median weight in kg (Q_1_=; Q_3_=)	69 (Q_1_ = 61; Q_3_ = 77)	60 (Q_1_ = 54; Q_3_ = 67)	
BMI			
Underweight	1 (1)	11 (7)	<0.05
Normal	22 (15)	10 (7)	
Overweight	76 (50)	107 (70)	
Obese	52 (34)	24 (16)	
Waist circumference (Q_1_=; Q_3_=)	78 (Q_1_ = 71; Q_3_ = 86)	75 (Q_1_ = 71; Q_3_ = 82)	

### Socio-demographic Factors Associated with Uncontrolled Hypertension

Earning more than US$200 (United States dollars), smoking cigarettes, taking alcohol and adding salt to dishes at the time of eating were significant risk factors for uncontrolled hypertension. Being female, walking and cycling regularly and doing active leisure activities were significantly protective against uncontrolled hypertension (Table 4).

**Table 4:** Socio-demographic factors associated with uncontrolled hypertension among hypertensive clients on ART, Kadoma City, 2016

Variable		Uncontrolled hypertension		OR(95% CI)
		Yes n(%)	No n(%)	
Age	≥40	123 (81)	113 (74)	1.46 (0.85–2.52)
	<40	29 (19)	39 (26)	
Sex	female	94 (62)	110 (72)	0.62 (0.38–1.00)
	male	52 (38)	42 (28)	
Level of education	none	3 (2)	1 (1)	3.04 (0.31–29.56)
	some	149 (98)	151 (99)	
Type of occupation	manual	80 (53)	87 (57)	0.83 (0.53–1.31)
	sedentary	72 (47)	65 (43)	
Average income	≥200	61 (40)	44 (29)	1.65 (1.02–2.65)
	<200	91 (60)	108 (71)	
Smoking cigarettes	yes	37 (24)	15 (10)	2.94 (1.54–5.62)
	no	115 (76)	137 (90)	
Drinking any alcohol	yes	39 (39)	17 (22)	2.20 (1.33–3.64)
	no	113 (74)	118 (78)	
Drinking sorghum beer	yes	44 (29)	27 (18)	1.89 (1.09–3.25)
	no	108 (71)	125 (82)	
Drinking wine	yes	20 (13)	8 (5)	2.73 (1.16–6.40)
	no	132 (87)	144 (95)	
Drinking lagers	yes	57 (38)	26 (17)	2.91 (1.70–4.96)
	no	97 (62)	126 (83)	
Adding salt to dishes	yes	123 (81)	60 (39)	6.50 (3.87–10.93)
	no	29 (19)	92 (61)	
Commonly used transport	walking and cycling	106 (70)	126 (83)	0.48 (0.28–0.82)
	driving	46 (30)	26 (17)	
Leisure activities	active	87 (57)	105 (69)	0.50 (0.37–0.96)
	passive	65 (43)	47 (31)	

### Medical Factors Associated with Uncontrolled Hypertension

The significant medical risk factors for uncontrolled hypertension among clients on ART were; having BMI above 25 kg/m2, a history of elevated hypertension in the previous year, taking anti-hypertensive medicines in the preceding 2 weeks and a family history of hypertension. A waist circumference <75 cm and having Stavudine/Lamivudine/Nevirapine as first regimen were significantly protective against uncontrolled hypertension (Table 5).

**Table 5:** Medical factors associated with uncontrolled hypertension among hypertensive clients on ART, Kadoma City, 2016

Variable		Uncontrolled hypertension		OR(95% CI)
		Yes n(%)	No n(%)	
BMI	≥25	75 (49)	34 (22)	3.38 (2.06–5.55)
	<25	77 (51)	118 (78)	
Waist circumference	<75 cm	10 (7)	14 (9)	0.60 (0.38–0.95)
	≥75 cm	142 (93)	138 (91)	
Duration since hypertension diagnosis	<5	95 (63)	108 (71)	0.68 (0.42–1.10)
	≥5	57 (37)	44 (29)	
Being on hydrochlorothiazide	yes	100 (66)	114 (75)	0.64 (0.39–1.05)
	no	52 (34)	38 (25)	
Being on amlodipine	yes	12 (8)	2 (1)	6.43 (1.41–29.23)
	no	140 (92)	150 (99)	
Elevated blood pressure in last year	yes	87 (57)	40 (26)	3.75 (2.31–6.08)
	no	65 (43)	112 (74)	
Taking anti-hypertensives in last 2 weeks	yes	114 (75)	95 (63)	1.80 (1.09–2.96)
	no	38 (25)	57 (37)	
Always having anti-hypertensives	yes	112 (74)	94 (62)	1.73 (1.06–2.81)
	no	40 (26)	58 (38)	
Duration since HIV diagnosis	≥2	79 (52)	91 (60)	0.73 (0.46–1.14)
	<2	73 (48)	61 (40)	
Duration on ART	<2	80 (53)	89 (59)	0.79 (0.59–1.24)
	≥2	72 (47)	63 (41)	
Current CD4+ count	<500	57 (43)	61 (46)	0.87 (0.54–1.42)
	≥500	76 (57)	71 (54)	
Currently on Tenolam/Efavirenz	yes	144 (95)	144 (95)	1.00 (0.37–2.74)
	no	8 (5)	8 (5)	
Currently on Tenolam/Atazanavir	yes	5 (3)	6 (4)	0.83 (0.25–2.77)
	no	147 (97)	146 (96)	
Previously on Stalanev	yes	23 (15)	37 (24)	0.55 (0.31–0.99)
	no	129 (85)	115 (76)	
Previously on Tenolam/Nevirapine	yes	30 (20)	18 (12)	1.83 (0.97–3.45)
	no	124 (80)	134 (88)	
Family history of hypertension	yes	108 (71)	91 (60)	1.65 (1.02–2.65)
	no	44 (29)	61 (40)	
Family history of stroke	yes	24 (16)	14 (9)	1.85 (0.92–3.73)
	no	128 (84)	138 (91)	

### Independent factors associated with uncontrolled hypertension

Adding salt to dishes aOR = 5.69 (3.19–10.16), BMI ≥ 25 kg/m2 aOR = 2.81 (1.60–4.91) and history of elevated blood pressure in previous year aOR = 2.34 (1.33–4.13) were independent risk factors. Independent protective factors were duration ≥2 years since HIV diagnosis aOR = 0.58 (0.35–0.95), duration <5 years since hypertension diagnosis aOR = 0.50 (0.30–0.83) and walking or cycling as a means of transport aOR = 0.27 (0.16–0.48).

## Discussion

Adding salt to dishes at time of eating was positively associated with uncontrolled hypertension. Clients who habitually add salt to their dishes have a higher salt content in their dishes. Salt in the body is associated with water retention resulting in high intravascular volumes. This adds the effort that the heart puts into pumping of blood resulting in hypertension. As a result hypertension control becomes poor [21]. This is consistent with a study by Mutede et al. [1] and studies in the general population concluded that high salt intake was associated with uncontrolled hypertension [16–18].

Clients doing well on ART are no longer susceptible to opportunistic infections that cause malabsorption syndromes. While weight gain in a client on ART is a good sign of responding to therapy, there is need for close monitoring. High BMI due to sustained weight gain is associated with metabolic syndrome. Metabolic syndrome is a precursor to conditions such as diabetes mellitus and hypertension due to insulin resistance [5, 6]. This finding is consistent with studies by Thiebaut et al. [22], Baekken et al. [23], Malaza et.al [24] and De Socio et al. [25] who reported a positive association between hypertension and higher BMI. This presents the need for on-going dialogue with clients to encourage healthy lifestyles and avoiding excess weight gain.

Duration since HIV diagnosis ≥2 years was negatively associated with uncontrolled hypertension. This is consistent with De Socio et al. [25]. Immediately after diagnosis of HIV, clients have to deal with psychological issues that come about because of having an incurable condition. With time, they start to accept the condition and ‘live positively’ resulting in better control of the hypertension. It then becomes imperative that the clients receive psychological support to ensure early acceptance and good control of hypertension.

Having less than 5 years after diagnosis of hypertension was negatively associated with uncontrolled hypertension. This result is consistent with Yameogo et al [26]. Having good blood pressure control early on could be as a result of the fact that the clients will have just received the information and are still very aware of the adverse effects of the condition that led to them seeking treatment. This therefore means they are more likely to be adherent to therapy and lifestyle changes. With time, they become used to the condition and are no longer ‘worried’ so they then tend to forget. It is therefore important to encourage adherence to medicines and lifestyle changes.

We also found that those who always had their anti-hypertensive medicines and those who reported having taken their medicines in the preceding 2 weeks were more likely to have uncontrolled hypertension. Always having medicines implies good access to them and would be expected to result in the taking of the medicines and good control of hypertension. It is possible that the clients who had the medicines were not necessarily taking them. This could be because of side effects or not perceiving the medicines to be important. At the same time, medicine dosages may have been inadequate. This is an area that was not adequately addressed in this study and would require further investigation.

‘White coat hypertension’ cannot be ruled out since this was health centre based. Some of the variables were recorded basing on the information reported by the clients. Information bias cannot be ruled out. No laboratory investigations were done due to financial constraints and this a limitation in this study.

## Conclusions

Having no education, being above 40 years of age, smoking, drinking alcohol (sorghum beer, lagers and wines), and adding salt regularly to dishes were socio-demographic risk factors for uncontrolled hypertension among hypertensives on ART. Being female, doing manual work, walking and cycling were socio-demographic protective factors against uncontrolled hypertension. The significant medical risk factors for uncontrolled hypertension among clients on ART were; having BMI above 25 kg/m^2^, a history of elevated hypertension in the previous year, taking anti-hypertensive medicines in the preceding 2 weeks and a family history of hypertension. Having a waist circumference less than 75 cm and having Stavudine/Lamivudine/Nevirapine as the first regimen were significant protective factors against uncontrolled hypertension.

Adding salt to dishes, a history of elevated blood pressure in the previous year, BMI > 25, duration since HIV diagnosis ≥2 years, duration since hypertension diagnosis <5 years and walking or cycling as a means of transport were independently associated with uncontrolled hypertension. Health education on lifestyle changes like walking and cycling as transport and dietary modification such as salt intake reduction were recommended.

## Abbreviations

ART: Anti-retroviral therapy
ARV: Anti-retroviral
BMI: Body mass index
BP: Blood pressure
CD4: Cluster of differentiation
CDC: Centers for Disease Control and Prevention
DALYS: Disability adjusted life years
GLUT: Glucose transporter
HIV: Human immunodeficiency virus
IHTC: Integrated HIV and Tuberculosis Clinic
JNC 7: Seventh Report of the Joint National Committee on the Prevention, Detection, Evaluation, and Treatment of High Blood Pressure
JREC: Joint Research Ethics Committee for the University of Zimbabwe, College of Health Sciences and the Parirenyatwa Group of Hospitals
MRCZ: Medical Research Council of Zimbabwe
WHO: World Health Organisation
